# Fatigue Perception in Adolescents with Obesity and Their Caregivers

**DOI:** 10.3390/jcm12134268

**Published:** 2023-06-26

**Authors:** Anna Guerrini Usubini, Michela Bottacchi, Adele Bondesan, Nicoletta Marazzi, Gianluca Castelnuovo, Alessandro Sartorio

**Affiliations:** 1 Psychology Research Laboratory, Istituto Auxologico Italiano, Istituto di Ricovero e Cura a Carattere Scientifico (IRCCS), 20145 Milan, Italy; m.bottacchi@auxologico.it (M.B.); gianluca.castelnuovo@auxologico.it (G.C.); 2 Experimental Laboratory for Auxo-Endocrinological Research, Istituto Auxologico Italiano, Istituto di Ricovero e Cura a Carattere Scientifico (IRCCS), 28824 Piancavallo-Verbania, Italy; a.bondesan@auxologico.it (A.B.); sartorio@auxologico.it (A.S.); 3 Experimental Laboratory for Auxo-Endocrinological Research, Istituto Auxologico Italiano, Istituto di Ricovero e Cura a Carattere Scientifico (IRCCS), 20145 Milan, Italy; n.marazzi@auxologico.it; 4 Department of Psychology, Catholic University of Milan, 20123 Milan, Italy

**Keywords:** childhood obesity, adolescents, fatigue, quality of life, parents

## Abstract

Although fatigue is a frequently occurring symptom in young patients with obesity, relatively few studies have assessed their perception of fatigue and its impact on their quality of life so far. Comparisons between the reports of fatigue in children/adolescents with obesity and their parents were assessed using the Pediatric Quality of Life Inventory-Multidimensional Fatigue Scale (PedsQL-MFS). One hundred Italian children/adolescents (36 males; 64 females), aged between 11 and 17 (mean age = 15.3; SD = 1.61) with severe obesity [mean Body Mass Index (BMI: kg/m^2^) = 38; SD = 5.48] and their caregivers were enrolled. Perception of fatigue did not change by sex and rates of obesity in adolescents, while there was a difference (*p* = 0.040) in parents’ reports of cognitive fatigue between parents of children/adolescents of Group 1 (BMI SDS 2–2.99) and Group 2 (BMI SDS > 3), with a higher perception of fatigue in parents of the less heavy obese children. Significant differences in reports of general fatigue subscale were found between children/adolescents and their parents, being higher in their parents than in the young subjects with obesity (*p* < 0.001). Significant moderate correlations between all the subscales of PedsQL-MFS for children and parents were found with Pearson’s coefficients ranging from 0.529 to 0.571 (*p* < 0.001). The perception of fatigue measured with the PedsQL-MFS was comparable between obese children and their parents, thus indicating that this symptom is not hidden by children and is clearly perceived by their parents.

## 1. Introduction

The World Health Organization recently estimated that the prevalence of obesity nearly tripled over the last forty years, worldwide. Over 340 million children and adolescents aged 5–19 were overweight or obese in 2016, and 38.2 million children under the age of 5 years were overweight or obese in 2019 [1]. 

Childhood obesity is associated with several comorbidities, such as type 2 diabetes, insulin resistance, other metabolic diseases, cardiovascular and respiratory diseases (such as obstructive sleep apnoea), and musculoskeletal problems. However, the effects of childhood obesity are not only restricted to physical health, but they have an impact on psychological functioning [2], as childhood obesity severely impacts all the domains composing the so-called health-related quality of life. Obesity in children and adolescents is also associated with low self-esteem, poor body image [3], depression [4], and decreased cognitive performance [5]. Obese children are often bullied and face numerous social consequences, such as victimization, stigmatization, and marginalization [6].

Fatigue is reported as an important psychological aspect for a significant number of children and adults affected by different clinical conditions [7], including obesity [8]. Fatigue has been defined as an extreme and persistent feeling of tiredness, weakness, or exhaustion that is experienced during physical or intellectual activities and is not reduced or alleviated by resting [9]. In obesity, fatigue is usually associated with psychological distress and disability, and, importantly, it negatively interferes with therapeutic physical activities, making them more difficult to be performed and also reducing diet adherence [10]. Although fatigue represents a crucial factor in conditioning the course of obesity, little research has been conducted evaluating the relationships between fatigue and obesity in children/adolescents with obesity so far. 

Pediatric obesity and fatigue have been previously associated [8], although the nature and direction of such associations were not entirely clear. One possible explanation is that people with higher levels of fatigue may be more susceptible to increasing BMI via less physical activity and a more sedentary lifestyle [11]. In addition, taking into account the link between pediatric and adult obesity, the evaluation of fatigue in pediatric obesity becomes essential to monitor the progression of the disease, its impact on quality of life, and treatment compliance [12]. Despite the well-known link between fatigue and obesity, relatively few studies have objectively assessed patients’ perceptions of fatigue in their life. 

The Pediatric Quality of Life Inventory-Multidimensional Fatigue Scale (PedsQL-MFS) [12] is the most used tool to investigate fatigue in pediatric age, assessing fatigue in the cognitive, physical, and sleep/rest dimensions [13]. One of the most interesting aspects of PedsQL-MFS is that it is designed to be administered as a child self-report and, simultaneously, as a parent proxy report instrument, offering the opportunity to be informed about fatigue from different points of view. For this reason, it should be considered a valid tool to be adopted for studying fatigue in pediatric obesity. It is a promising research area in which less effort has been made to investigate together the child and parent reports about the perception of fatigue in pediatric obesity. Most of the current studies assessing fatigue in children and adolescents with obesity involved only the self-report form of PedsQL-MSF [14], while, on the other hand, parental perceptions in the field of pediatric obesity were primarily assessed only via qualitative studies [15].

The objective of the present study is to describe the perception of fatigue in children/adolescents with obesity and their parents, comparing the two versions of the Pediatric Quality of Life Inventory-Multidimensional Fatigue Scale (PedsQL-MFS). 

## 2. Materials and Methods

### 2.1. Participants and Procedures 

This was a cross-sectional study on the association between obesity and a variety of adolescent outcomes, including cognitive fusion, emotional eating [2], and fatigue perception. One hundred and five Italian adolescents (38 males; 67 females) aged between 11 and 17 (mean age = 15.3; SD = 1.61) with severe obesity [mean Body Mass Index (BMI) = 38; SD = 5.48] and their caregivers were initially screened for the participation at the study. Obesity was defined as having a BMI standard deviation score (BMI SDS) for sex and chronological age greater than 2, according to the Italian growth charts [16]. Young patients and/or their caregivers with cognitive impairment and unable to understand the questionnaire were excluded (*n* = 5). After completing both informed consent by parents and written assent to participate by children, participants were asked to complete a self-report questionnaire. The questionnaire was self-administered. Children and their parents completed the questionnaire separately. The current study was approved by the Ethical Committee of Istituto Auxologico Italiano, IRCCS, Milan, Italy (research project code: 01C625; acronym: FATIPSICOB). The research was carried out according to the Declaration of Helsinki and its advancements.

### 2.2. Measures 

The children and parent versions of the Pediatric Quality of Life Inventory-Multidimensional Fatigue Scale (PedsQL-MFS) [12,17], a self-report questionnaire consisting of 18 items describing symptoms of fatigue. Each item is rated on a 5-point Likert scale from 0 “almost never” to 4 “almost always”. Italian child report versions for 8–12 year-olds and 13–18 year-olds previously validated for use in pediatric obesity by our group were used. PedsQL-MFS comprises three subscales: cognitive fatigue exploring difficulties in cognitive functions (i.e.,“it is hard for me to maintain attention on things”; “it is hard for me to remember what people tell me”), sleep/rest fatigue assessing sleep disorders (i.e., “I feel tired when I wake up in the morning”; “I rest a lot”), and general fatigue assessing the general perception of fatigue during the day (i.e., “I feel tired”; “I feel too tired to do things that I like to do”).

### 2.3. Statistical Analysis 

Sample size calculation for mean comparisons was performed using G*Power software (release 3.1.9.4).

Setting alpha to 0.05 and power to 0.80, a total sample of 200 (100 children and 100 parents) was considered sufficient to detect a small-to-medium effect size (d = 0.40).

Descriptive statistics were computed for all demographic, physical, and clinical variables. The normal distribution of the variables was assessed by skewness and kurtosis indices. Differences between males and females, as well as different rates of obesity (Group 1: BMI SDS 2–2.99 vs. Group 2: BMI SDS > 3) and differences in PedsQL-MFS parent versions according to sex and rates of obesity of their children, were evaluated using independent sample *t*-tests. Relations between parents and children versions of PedsQL-MFS subscales were also analyzed with Pearson’s correlation. Critical alpha was set to 0.05.

All analyses were performed with Jamovi (The jamovi project 2021). Jamovi (Version 2.3.2) [Computer Software] retrieved from https://www.jamovi.org. 

## 3. Results

### 3.1. Description of the Sample 

In this study, there were no missing data. From the initial screening, five participants were excluded since they did not meet our inclusion/exclusion criteria. The final sample was composed of one hundred Italian adolescents (36 males; 64 females), aged between 11 and 17 (mean age = 15.3; SD = 1.61) with severe obesity [mean Body Mass Index (BMI) = 38; SD = 5.48], and their parents. Descriptive statistics of the sample are presented in Table 1. Most of the participants had a secondary school degree (78%). All the subscales of PedsQL-MFS of both versions had a good-to-high internal consistency (α ranged from 0.727 to 0.920). Table 2 shows means and standard deviations for PedsQL-MFS subscales of both versions by sex and rates of obesity of children/adolescents and their comparisons. Results showed no statistically significant differences between reports of children/adolescents according to their sex and rate of obesity (group 1: BMI SDS: 2–2.99; group 2: BMI SDS: > 3).

Similarly, no differences in parents’ reports of PedsQL-MFS based on sex and rates of obesity of their children were found, except for cognitive fatigue (*p* = 0.040) which was higher in parents of children/adolescents of group 1 than group 2. 

### 3.2. Comparison between the Perceptions of Fatigue in Children/Adolescents vs. Their Parents

In order to compare the perceptions of fatigue in children/adolescents with obesity vs. their parents, independent sample *t*-tests were run. Results are presented in Table 3. Significant differences between children’s and parents’ reports in general fatigue (*p* < 0.001) and total fatigue (*p* = 0.002) were found, with higher means of both subscales being recorded in parents rather than in their children/adolescents. By contrast, no significant differences in the reports of sleep–rest and cognitive fatigue were found between children/adolescents and their parents. 

Beyond specific differences, similar trends were observed in perceptions of fatigue in children/adolescents with obesity and their parents, as demonstrated by moderate but significant Pearson’s correlations between all the subscales of PedsQL-MFS, with coefficients ranging from 0.529 to 0.571 (*p* < 0.001). 

## 4. Discussion

Fatigue is a debilitating condition that aggravates the impact of obesity on HRQOL and mental health [8]. Despite its recognized effects, there is a scarcity of studies addressing the perception of fatigue in young obese individuals and their parents. Our study quantitatively assessed fatigue in a pediatric sample suffering from obesity and their parents, comparing the two versions of PedsQL-MFS. 

According to our results, the perception of fatigue did not change by sex and rates of obesity in adolescents, suggesting that fatigue is a common health-related condition associated with pediatric obesity. The lack of gender-related differences in fatigue perceptions has not yet been extensively studied in the obese population, but finds partial confirmation in the literature. Lassandro and colleagues [18] studied the perception of fatigue in young patients with chronic immune thrombocytopenia and their caregivers via PedsQL-MFS and no statistically significant differences in fatigue perception between the two genders were found, thus indicating the predominant role of the disease per se. Similar results were obtained by Ghajarzadeh and colleagues [19], who found that fatigue in multiple sclerosis was not related to gender but was influenced by other factors, such as the duration of the disease. 

As far as parents’ reports are concerned, our results did not show statistically significant differences according to the child’s sex and rate of obesity, with the only exception of cognitive fatigue which was higher in parents of obese children with lower BMI.

Although our study allowed us to assess possible differences in the perception of fatigue in relation to the different degrees of obesity (measured by BMI SDS), it does not actually take into account additional factors that could have an impact on the perception of fatigue, such as the duration of the disease and/or the individual psychological status, and, therefore, it will require further additional studies for definitive confirmation.

Comparisons between children/adolescents and their parents’ reports showed similar results, with no significant differences between children/adolescents and their parents across sleep/rest and cognitive fatigue. However, parents showed a higher perception of fatigue in their children with respect to general fatigue (i.e., “*I feel physically weak*”) compared to their sons or daughters. 

Correlations between PedsQL-MFS of children and parents showed moderate significant associations between reports of young patients and those of their caregivers, suggesting that the lower the level of fatigue perception in children, the lower the level of fatigue in parents’ perceptions and vice-versa. Such results were in line with previous findings suggesting similar perceptions of fatigue in children and their caregivers in chronic conditions [20]. Our results are also in line with previous data collected by our research team in the field of psychological adjustment of children/adolescents with obesity in which parents/children agreement was evaluated with the Child Behavior Checklist (CBCL) for parents and Youth Self-Report (YSR) for children (under review). Our results showed positive and significant correlations between CBCL and YSR, thus suggesting a similar trend between children’s and parents’ perceptions. 

The present study has several limitations. The cross-sectional nature of the study warrants caution in making causal conclusions about the relationships between fatigue and obesity. Data available for analysis are limited to those collected in a single clinical center, and are, therefore, less generalizable to the entire population. In addition, there is a lack of demographic data about parents such as sex, age, socio-economic status, or other clinical data that could influence the perception of their child and should be considered for the study. Finally, the fact that we obtained future replications of this work will take into consideration additional data from children and their parents. Furthermore, despite the agreement in the reports by children and parents, we are unable to definitively detect the ability of parents to perceive the child condition independently from the quality of their relationship. Despite limitations, the study presents also some strengths. The study employed a measure of fatigue that has been validated in pediatric obese populations. The sample was relatively large for a clinical population and included participants of both genders with a severe degree of obesity.

To address limitations and provide further knowledge about fatigue in youth obesity, advancements of this study should include additional socio-demographic information, including other clinical centers, and make comparisons with a control group of normal-weight adolescents, as well as including longitudinal measures to detect eventual changes over time of fatigue. In addition, future directions of the study should be moved toward the implementation of treatments addressing fatigue in obese populations.

In conclusion, the perception of fatigue measured with the PedsQL-MFS was comparable between obese children and their parents, thus indicating that, although this symptom can be considered less visible [20], it is clearly perceived by parents of youth with obesity. Since fatigue in pediatric obesity impacts heavily quality of life, ability to perform physical activity, and adherence to diets, the adequate perception by parents of sleep/rest fatigue, cognitive fatigue, and general fatigue in their children with obesity must represent a further stimulus to embark on a multidisciplinary body weight reduction program for their sons/daughters. 

## Figures and Tables

**Table 1:** Descriptive statistics of the sample.

		*n*	Mean (SD)	α
Sex	Males	36		
	Females	64		
Age			15.3 (1.61)	
Educational level	Primary school	22		
	Secondary school	78		
BMI			38 (5.48)	
BMI SDS			3.05 (0.51)	
PedsQL.MFS child	Cognitive fatigue	100	6.49 (4.98)	0.870
	Sleep/rest fatigue	100	7.98 (4.73)	0.727
	General fatigue	100	7.34(4.11)	0.809
	Total fatigue	100	21.81 (11.14)	0.878
PedsQL_MFS parent	Cognitive fatigue	100	7.83 (5.66)	0.920
	Sleep/rest fatigue	100	9.00 (5.43)	0.868
	General fatigue	100	10.25 (4.57)	0.789
	Total fatigue	100	27.08 (12.86)	0.913

**Table 2:** Means (SD) and Student’s t (*p*) of PedsQL-MFS child and parent versions between sex and rates of obesity of children.

			*n*	Mean (SD)	Student’s t (*p*)	*p*
PedsQL-MFS child	Cognitive fatigue	Males	36	6.06 (4.64)	−0.652	0.516
		Females	64	6.73/5.18)		
		BMI SDS 2–2.99	45	7.40 (5.13)	1.667	0.099
		BMI SDS > 3	55	5.75 (4.78)		
	Sleep/rest fatigue	Males	36	7.06 (4.55)	−1.475	0.143
		Females	64	8.50/4.78)		
		BMI SDS 2–2.99	45	7.98 (4.60)	−0.004	0.997
		BMI SDS > 3	55	7.98 (4.87)		
	General fatigue	Males	36	6.44 (4.30)	−1.649	0.102
		Females	64	7.84 (3.94)		
		BMI SDS 2–2.99	45	7.89 (4.16)	1.211	0.229
		BMI SDS > 3	55	6.89 (4.04)		
	Total fatigue	Males	36	19.56 (10.73)	−1.528	0.130
		Females	64	23.08 (11.24)		
		BMI SDS 2–2.99	45	23.27 (10.92)	1.185	0.239
		BMI SDS > 3	55	20.62 (11.27)		
PedsQL-MFS parent	Cognitive fatigue	Males	36	7.92 (5.63)	0.114	0.909
		Females	64	7.78 (5.72)		
		BMI SDS 2–2.99	45	9.11 (5.43)	2.082	0.040 *
		BMI SDS > 3	55	6.78 (5.67)		
	Sleep/rest fatigue	Males	36	8.50 (5.06)	−0.689	0.492
		Females	64	9.28 (5.64)		
		BMI SDS 2–2.99	45	9.00 (4.94)	0.000	1.000
		BMI SDS > 3	55	9.00 (5.84)		
	General fatigue	Males	36	9.28 (4.78)	−1.608	0.111
		Females	64	10.80 (4.39)		
		BMI SDS 2–2.99	45	10.29 (4.58)	0.076	0.939
		BMI SDS > 3	55	10.22 (4.61)		
	Total fatigue	Males	36	25.69 (12.30)	−0.807	0.422
		Females	64	27.86 (13.20)		
		BMI SDS 2–2.99	45	28.40 (11.90)	0.927	0.356
		BMI SDS > 3	55	26.00 (13.61)		

Note: * *p* < 0.05.

**Table 3:** Comparisons and correlations between child and parent versions of PedsQL-MFS.

		Student’t (*p*)	Pearson’s *r* (*p*)
Cognitive fatigue child	Cognitive fatigue parent	−1.78 (0.077)	0.571 (*p* < 0.001)
Sleep/rest fatigue child	Sleep/rest fatigue parent	−1.42 (0.158)	0.529 (*p* < 0.001)
General fatigue child	General fatigue parent	−4.74 (<0.001)	0.531 (*p* < 0.001)
Total fatigue child	Total fatigue parent	−3.10 (0.002)	0.557 (*p* < 0.001)

## Data Availability

Raw data will be uploaded on www.Zenodo.org immediately after the acceptance of the manuscript and they will be available upon a reasonable request to the authors A.G.U. and A.S.

## References

[1] World Health Organization (2022). WHO European Regional Obesity Report 2022.

[2] Guerrini Usubini A., Bottacchi M., Caroli D., Castelnuovo G., Sartorio A. (2022). Cognitive Fusion and Emotional Eating among Adolescents with Obesity: A Preliminary Cross-Sectional Study. Int. J. Environ. Res. Public Health.

[3] Gow M., Tee M., Garnett S., Baur L., Aldwell K., Thomas S., Lister N., Paxton S., Jebeile H. (2020). Pediatric obesity treatment, self-esteem, and body image: A systematic review with meta-analysis. Pediatr Obes..

[4] Wardle J., Williamson S., Johnson F., Edwards C. (2005). Depression in adolescent obesity: Cultural moderators of the association between obesity and depressive symptoms. Int. J. Obes..

[5] Li Y., Dai Q., Jackson J.C., Zhang J. (2008). Overweight Is Associated with Decreased Cognitive Functioning among School-age Children and Adolescents. Obesity.

[6] Sahoo K., Sahoo B., Choudhury A.K., Sofi N.Y., Kumar R., Bhadoria A.S. (2015). Childhood obesity: Causes and consequences. J. Fam. Med. Prim. Care.

[7] Hockenberry M.J., Hinds P.S., Barrera P., Bryant R., Adams-McNeill J., Hooke C., Rasco-Baggott C., Patterson-Kelly K., Gattuso J.S., Manteuffel B. (2003). Three Instruments to Assess Fatigue in Children with Cancer: The Child, Parent and Staff Perspectives. J. Pain Symptom Manag..

[8] Norris T., Hawton K., Hamilton-Shield J., Crawley E. (2016). Obesity in adolescents with chronic fatigue syndrome: An observational study. Arch. Dis. Child..

[9] Singh B., Negatu M.G., Francis S.L., Janz K.F., Yack H.J. (2016). Do fitness and fatigue affect gait biomechanics in overweight and obese children?. Gait Posture.

[10] Mostafavi-Darani F., Zamani-Alavijeh F., Mahaki B., Salahshouri A. (2020). Exploring the barriers of adherence to dietary recommendations among patients with type 2 diabetes: A qualitative study in Iran. Nurs. Open.

[11] Newton J.L., Pairman J., Hallsworth K., Moore S., Plotz T., Trenell M.I. (2011). Physical activity intensity but not sedentary activity is reduced in chronic fatigue syndrome and is associated with autonomic regulation. QJM Int. J. Med..

[12] Manzoni G.M., Rossi A., Marazzi N., Agosti F., De Col A., Pietrabissa G., Castelnuovo G., Molinari E., Sartorio A. (2018). Feasibility, Validity, and Reliability of the Italian Pediatric Quality of Life Inventory Multidimensional Fatigue Scale for Adults in Inpatients with Severe Obesity. Obes. Facts.

[13] Varni J.W., Seid M., Rode C.A. (1999). The PedsQLTM: Measurement model for the pediatric quality of life inventory. Med. Care.

[14] Smout M., Manzoni G.M., Tamini S., Marazzi N., De Col A., Pietrabissa G., Castelnuovo G., Molinari E., Sartorio A. (2022). Pediatric quality of life multidimensional fatigue scale (PedsQL-MFS) detects the effects of a 3-week Inpatient body weight reduction program for children and adolescents with obesity. Health Qual. Life Outcomes.

[15] Pocock M., Trivedi D., Wills W., Bunn F., Magnusson J. (2010). Parental perceptions regarding healthy behaviours for preventing overweight and obesity in young children: A systematic review of qualitative studies. Obes. Rev..

[16] Cacciari E., Milani S., Balsamo A., Spada E., Bona G., Cavallo L., Cerutti F., Gargantini L., Greggio N., Tonini G. (2006). Italian Cross Sectional Growth Charts for Height, Weight and BMI (2 to 20 yr). J. Endocrinol. Investig..

[17] Mapi Res Institute (2002). Linguistic Validation of the PedsQL—A Quality of Life Questionnaire: Research and Evaluation, Limited Use Translation of PedsQL.

[18] Lassandro G., Palmieri V.V., Barone A., Farruggia P., Giona F., Licciardello M., Marinoni M., Marzollo A., Notarangelo L.D., Palumbo G. (2021). Fatigue perception in a cohort of children with chronic immune thrombocytopenia and their caregivers using the PedsQL MFS: Real-life multicenter experience of the Italian Association of Pediatric Hematology and Oncology (AIEOP). Pediatr. Blood Cancer.

[19] Ghajarzadeh M., Jalilian R., Eskandari G., Sahraian M.A., Azimi A., Mohammadifar M. (2013). Fatigue in multiple sclerosis: Relationship with disease duration, physical disability, disease pattern, age and sex. Acta Neurol. Belg..

[20] Charvet L.E., Kluzer B., Krupp L.B. (2014). Invisible Symptoms of MS: Fatigue, Depression, and Cognition. Multiple Sclerosis and CNS Inflammatory Disorders.

